# Mediterranean diet intervention among World Trade Center responders with post‐traumatic stress disorder: Feasibility and outcomes of a pilot randomized controlled trial

**DOI:** 10.1002/osp4.725

**Published:** 2023-12-21

**Authors:** Chrisa Arcan, Wei Hou, Kathryn Hoffman, Amanda Reichardt, Xiaohua Yang, Sean A. P. Clouston, Evelyn J. Bromet, Benjamin Luft

**Affiliations:** ^1^ Department of Epidemiology School of Population Health Virginia Commonwealth University Richmond Virginia USA; ^2^ Department of Family Population and Preventive Medicine Renaissance School of Medicine Stony Brook University Stony Brook New York USA; ^3^ Vertex Pharmaceuticals Incorporated Boston Massachusetts USA; ^4^ Stony Brook World Trade Center Health Plan and Wellness Program Renaissance School of Medicine Stony Brook University Commack New York USA; ^5^ Neurosciences Institute Renaissance School of Medicine Stony Brook University Stony Brook New York USA

**Keywords:** body mass index, Mediterranean diet, post traumatic stress disorder, systemic inflammation, waist circumference, WTC responders

## Abstract

**Objective:**

Responders of the World Trade Center (WTC) disaster suffer from co‐morbidities. A Mediterranean Diet (MedDiet) nutrition intervention with physical activity was implemented among WTC responders with overweight/obesity and post‐traumatic stress disorder (PTSD).

**Methods:**

WTC Health Program members (*N* = 62), 45–65 years, males 87%, body mass index (BMI) 27–45 kg/m^2^ randomized to MedDiet (*n* = 31) or usual nutrition counseling (*n* = 31). The 10‐week intervention included online nutrition education, text messages, and group experiential cooking; both groups had three in‐person individual nutrition counseling. Anthropometrics, serum biomarkers, psychosocial factors, MedDiet score, and PTSD symptoms were assessed at baseline, post‐intervention, and 3‐months (follow‐up). The primary outcome was intervention feasibility and secondary outcomes were within‐ and between‐group changes of all measures at post‐intervention and follow‐up. Nonparametric Wilcoxon rank sum tests for between‐group comparisons and Wilcoxon signed rank tests for pre‐post within‐group comparisons.

**Results:**

A total of 58(94%) and 46(74%) participants completed the post‐intervention and follow‐up measurements, respectively. Both groups experienced significant improvements in anthropometrics, MedDiet score, oxidized low‐density lipoprotein, and PTSD symptoms. Baseline median (range) were weight 100.42 (73.66–135.17) kg, BMI 33.20 (27.50–41.75) kg/m^2^, and Waist circumference (WC) 109.22 (90.17–150.62) cm. Median % weight loss at post‐intervention was MedDiet: −3% (−11%–7%), *p* = 0.0002; Control: −1% (−13%–4%), *p* = 0.008 and at follow‐up MedDiet: −2% (−14%–12%), *p* = 0.07; Control: −2% (−20%–3%), *p* = 0.006. The overall BMI was reduced by −0.68 kg/m^2^ (−4.61–2.09) kg/m^2^ *p* < 0.0001 at post‐intervention and by −0.60 kg/m^2^ (−6.91–3.39) kg/m^2^, *p* < 0.0009 at follow‐up. Overall, median WC was reduced (*p* < 0.0001); post‐intervention −3.81 cm (−33.00–3.30)cm and follow‐up −4.45(−38.10–4.57)cm. There were group differences in HbA1c (*p* = 0.019) and serum ω6/ω3 (*p* = 0.029) at post‐intervention.

**Conclusion:**

Online intervention with personal counseling was feasible in this population. Improvements in anthropometrics, MedDiet score, selected serum biomarkers and PTSD symptoms were found in both groups; group differences in HbA1c and serum ω6/ω3. A larger study with a delayed control is needed to better assess intervention effects.

## Graphical abstract

The World Trade Center disaster rescue workers and other responders continue to bear the burden of multiple co‐morbidities, including metabolic syndrome, cardiovascular disease, systemic inflammation, and post‐traumatic stress disorder (PTSD). This study describes the findings of a pilot randomized controlled trial assessing the feasibility of a nutrition intervention, focusing on the Mediterranean diet pattern to reduce weight and systemic inflammation among World Trade Center responders with overweight/obesity and PTSD. The study was feasible with high participant retention and satisfaction. Nutrition programming contributed to diet improvement, reduction of weight, waist circumference, body mass index, lipid and inflammatory biomarkers, and PTSD symptoms.

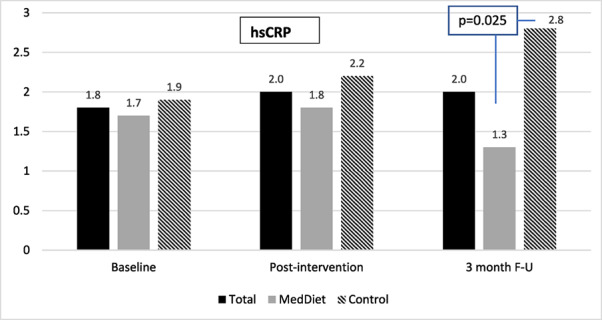

## 1 INTRODUCTION

Twenty years after the terrorist attacks of 11 September 2001 on the World Trade Center (WTC) in New York City, the rescue workers and other responders who helped with the clean‐up continue to bear the burden of multiple co‐morbidities mainly due to environmental exposure and psychological trauma.^1^, ^2^, ^3^ Trauma‐exposed WTC responders experience high prevalence of inflammation‐related conditions such as metabolic syndrome (MetS), cardiovascular disease, and chronic obstructive pulmonary disease.^1^ The average body mass index (BMI) among WTC responders is in the obesity range (i.e., >30 kg/m^2^)^4^ and nearly one‐third have at least three of the MetS factors with hypertension and abdominal obesity occurring most frequently.^5^ Post‐traumatic stress disorder (PTSD) is a psychiatric condition that can develop after a traumatic event and manifests in various psychological, biological, and behavioral outcomes^6^ that carry severe societal and economic consequences. One in five WTC responders who aided in the initial recovery operations suffer from PTSD.^7^

Evidence to date has shown the co‐occurrence of obesity and obesity‐related chronic diseases, such as type 2 diabetes, insulin resistance, and cardiovascular disease with PTSD.^8^, ^9^, ^10^ Moreover, individuals with PTSD have poorer dietary behaviors and have more difficulty achieving weight loss compared to individuals without PTSD.^11^, ^12^, ^13^ While the multimorbidity of obesity, MetS and PTSD^14^, ^15^ often poses challenges to medical interventions,^16^, ^17^ it also emphasizes the importance for lifestyle modification interventions incorporating diet and Physical Activity (PA) to reduce systemic inflammation, metabolic comorbidities, and weight in individuals with PTSD.^18^, ^19^

A systematic review of intervention trials involving anti‐inflammatory rich diets and exercise have shown to protect against low‐grade inflammation and attenuate inflammatory biomarkers.^20^ However, there is a scarcity of lifestyle modification interventions focusing on nutrition and PA among participants who suffer from PTSD.^21^ The Mediterranean Dietary pattern (MedDiet) is characterized by healthy dietary fats and antioxidant‐ and polyphenol‐rich foods derived mainly from plant‐based sources like fruits, vegetables, olive oil, beans, nuts, legumes, herbs, spices, and whole grains.^22^ In part, the benefits of a MedDiet are thought to emerge from its focus on meal combinations and foods that work synergistically to reduce postprandial inflammatory and oxidative risks.^23^, ^24^ Regular adherence to the MedDiet has been shown to have beneficial effects in primary and secondary prevention of cardiovascular disease, metabolic disorders, and breast cancer and to provide a neuroprotective effect.^19^, ^25^, ^26^, ^27^, ^28^ Emerging evidence points to the protective effect of the MedDiet through lipid reduction, protection against oxidative stress and inflammation, modification of hormone and growth factors linked to cancer pathogenesis, and gut microbiota influencing metabolic health.^29^

Lifestyle modification interventions delivered remotely utilizing the Internet and mobile technology have shown great promise in improving lifestyle behaviors including dietary intake, PA, and behavioral modification.^30^, ^31^, ^32^, ^33^ Here, we report results from the WTC‐Nutrition Study, a 10‐week prospective randomized control trial implementing the MedDiet with a PA component to improve eating behaviors among WTC responders with overweight and obesity and an active diagnosis of chronic PTSD. The intervention was mainly delivered using the Internet and a mobile technology with minimal in person counseling. This manuscript describes findings on study feasibility and on secondary outcomes, including anthropometrics, MedDiet score, PTSD symptoms (PCL score), and selected blood biomarkers.

## 2 MATERIALS AND METHODS

### 2.1 Study design and setting

The WTC‐Nutrition pilot study was a two‐arm randomized controlled trial conducted at the WTC Health Program (WTC‐HP) at Stony Brook University in Long Island, New York from December 2020 through November 2021. A total of 62 participants were randomized to intervention (MedDiet *n* = 31) and control (Usual care *n* = 31). The sample size was based on available budget and was also informed by other feasibility studies involving behavior change nutrition interventions and the home food environment.^34^, ^35^ The primary aim of the study was to assess the feasibility of the intervention and the secondary aims were that by the end of the intervention and at 3‐months post intervention (follow‐up) the MedDiet group compared to the control group would have had higher MedDiet score indicating higher intake of food representative of the Mediterranean dietary pattern, more favorable anthropometric measures, and improvements in lipid profile and inflammatory biomarkers.

The study participants were members of the WTC‐HP. In 2002, the Centers for Disease Control and Prevention established a monitoring and treatment program for 9/11 rescue and recovery workers. Stony Brook University has two WTC‐HP clinics and provides monitoring, treatment, and mental health services to a little more than 13,000 responders primarily residing on Long Island, NY. Overall, the participants at the program were mainly male (92%) and worked in law enforcement (71.5%) during the 9/11 disaster.^36^

### 2.2 Participant recruitment and screening

The screening and recruitment of study participants were conducted by the WTC‐HP registered dietitians who also served as study dietitians and by other clinic staff. Three recruitment methods were utilized: (1) in person during scheduled annual monitoring visits, (2) physician referrals for nutrition counseling within the past year, and (3) calling clinic members who met initial inclusion criteria and had signed prior informed consent agreeing to be contacted for future studies. The initial goal was to recruit participants with obesity (BMI ≥30 kg/m^2^), however due to the limited number of individuals meeting all the inclusion criteria, the decision was made to also include participants with BMI ≥27 kg/m^2^). Thus, the final inclusion criteria were BMI ≥27 to ≤40 kg/m^2^), age 40–65 years, diagnosed PTSD with a Checklist (PCL‐5) score ≥40 in the past year, and willingness to attend and provide a blood sample at baseline, follow‐up, and post‐intervention data collection visits. The specific age range was selected because the average age of WTC‐HP members was 53 years. Exclusion criteria included, active malignant cancer (except non‐melanoma skin cancer) and/or history of malignancy within the last three years for gastrointestinal‐related cancer, autoimmune disorders (associated with increased inflammatory markers), history of surgical weight loss, and evidence of cognitive impairment with Montreal Cognitive Assessment≤22 in the past year, previous or current nutrition counseling by WTC‐HP dietitians or participation in another lifestyle intervention, unwillingness or lack of ability to follow the recommended diet, physical difficulties that impair walking, inability to read and speak English, and not having a smartphone or unwilling to utilize text messaging services. All study procedures, including data collection and materials, were approved by Stony Brook University's Institutional Review Board. This trial was registered on clinicaltrials.gov NCT05138198 (11/29/2021).

Once recruitment was completed and participants signed the informed consent, a baseline meeting with the RDs was scheduled within 2 weeks in either of the two Stony Brook WTC‐HP clinics. One day prior to the meeting, participants received a reminder email, call, or a text message with an attachment of the “pre‐visit intake form” via Qualtrics to collect information on salient health behaviors, such as smoking, food preferences, readiness for behavioral change, medication and supplement use, and diabetes and mental health history; they also received instructions on how to download the Cronometer and Fitbit apps on their smart phones.

During the baseline visit that lasted about 60 min, the participants received information about study procedures, had their blood pressure, height, weight, and Waist circumference (WC) measured and their blood drawn by a clinic phlebotomist or a nurse; participants were instructed to be fasting from midnight to the time of their appointment. They received their personal Fitbit and were shown how to wear and sync it with their smartphones. Instructions on how to record their daily food intake on Cronometer app were also provided. Following the baseline visit, the participants received electronic surveys in batches via text messages with Qualtrics link to be completed over 1 week. The surveys included the 14‐question Mediterranean diet questionnaire, 26‐question Dietary Screener Questionnaire, and questions assessing the participants' nutrition knowledge, eating habits, weight history, social support, sleeping habits, and their home food environment.

After baseline measurements, the participants were randomized to MedDiet or control (usual nutrition counseling) groups using a computer‐generated randomization procedure. The participants were blinded to their study status. To promote adherence to measurements and retention, various incentives were provided, including a container of extra virgin olive oil (MedDiet only) or $25 gift card (control only) at baseline, $50 gift‐cards after each post‐intervention and follow‐up assessment, and $50 to cover transportation costs to/from the clinics. Participants were able to keep their Fitbit watch (Inspire HR model)^37^ as an incentive. After the group cooking session, a raffle was drawn, and one participant received a $50 air fryer.

### 2.3 Intervention modules (treatment arm: Mediterranean diet with physical activity tips)

The intervention adopted principles of Social Cognitive Theory,^38^ emphasizing behavior (healthy eating and PA) within the inclusion of social context (family/friend support) in a dynamic and reciprocal interaction between the participants and their environment (home food availability/family meals) and was informed by elements of the “Feeding America's Bravest” study.^39^ The three individual counseling meetings included addressing questions about the MedDiet, behaviors and habits, family support, anticipated barriers, and motivators.^39^, ^40^, ^41^

The WTC‐Nutrition intervention emphasized a higher consumption of foods rich in antioxidant and anti‐inflammatory components and lower consumption of foods with inflammatory potential. The goal was toward gradually increasing intake of a variety of vegetables, fruits, whole grains, seafood/fish, legumes, and white meat (poultry/turkey) and reducing the intake of red meat, processed meat, sugary beverages, chips, fried foods, and sweets. Extra virgin olive oil was recommended as the fat of choice.

The intervention was delivered using a multimodal approach to ensure high intervention dose by meeting the previously reported barriers to participation including the possibility of boredom and irregular work schedule.^5^ The main mode of intervention delivery was through the study website and mobile phones (text messages). Weekly content included a mix of psychoeducation and behavioral skills and strategies (e.g., goal setting, skills building and self‐efficacy to prepare and consume healthy meals, knowledge about the MedDiet, social support, mitigating barriers to change, and providing positive reinforcement). Each week the participants received a text message with the link to the study's website weekly session with content including a “food” and “fit” tips, recipes, and short videos with nutrition education and experiential skills development. Based on the theme/topic of each week, the participants received additional reminder and motivational text messages, as well as a text message with teach back questions following the videos and a prompt to set personal weekly goals. The weekly nutrition topics and activities are shown in Table 1. One live session of group experiential cooking was conducted remotely due to COVID‐19 imposed social isolation. Additionally, both the MedDiet and control groups received three in‐person counseling sessions during each of the measurement times.

**TABLE 1:** Overview of the WTC‐nutrition intervention weekly sessions.

Topics (Weeks)	Nutrition and PA themes
1. Introduction to the study: Live healthy to lose weight	Healthy habit building and meals and snacks to get started
	Setting realistic goals; importance of PA
	Family meals and social support
2. Med not meds	Introduction of MedDiet and increase minutes of exercise
	Introduction of olive oil and how to gradually increase consumption
3. Label lingo	Label reading education and portion control recommendations; guide to increase daily steps
4. Prep, plan, and save	Introduction to meal preparation and grocery store tour (virtual)
	Importance of making a grocery list, purchasing more produce (fruits, vegetables) and more seafood; PA choices to overcome challenges
5. Facts on fats and inflammation	Chronic inflammation education; sources for healthy fat intake
	Healthy fat sources: Nuts (including peanuts) and seeds
6. Eat the rainbow	Fighting inflammation with food and the importance of fruits and vegetables; fighting inflammation with PA
	Ways to increase intake of fruits and vegetables; consider challenges and opportunities
7. Bittersweet? Carbs, beverages and ways to reduce sugar intake	Carbohydrate education and importance of whole grains; importance of strength training
	Sugar‐sweetened beverages and simple carbs; ways to increase whole grains
8. How to balance a meal (emphasize olive oil for fat) and dining out Mediterranean + snacking & fast food	How to balance a meal and ways to dine out and snack healthfully
	Choose unsaturated fats over butter & cheese; choose grilled instead of fried options when dining; read the label or menu ahead of time
	Explore healthy food swaps for cravings when they arise
9. “Eat food, not a lot, mostly plants”	Importance of a plant‐based diet and plant‐based proteins in the diet
	Ways to increase meatless meals per week
10. Healthy for life	Tips for time management and sustainable habits
	Maintain my physical activity routine; be mindful about my food choices in various occasions; increase social support in eating and PA

*Note*: The intervention themes were delivered through a study website, motivational and reminder text messages, videos, goal setting (weekly).

Abbreviation: PA, Physical Activity.

### 2.4 Control group (usual care)

The control group received three in‐person individual counseling sessions with their assigned dietitian. The dietitian provided medical nutrition therapy, a specific application of the Nutrition Care Process in clinical settings that focuses on the management of diseases (e.g., diabetes, heart disease, etc.). Specific dietary recommendations were based on the Bull's Eye Food Guide used by Stony Brook University Hospital for outpatient nutrition counseling.^42^

### 2.5 Measures

Data collection occurred at three time points: baseline (11/2020–2/2021), post‐intervention (5/2021; 3 months from baseline), and follow‐up (8/2021; 6 months from baseline) to assess the sustainability of behaviors. Study feasibility was assessed through careful recording of participant recruitment and retention process, the completion of measurements in three time points, compliance of weekly video review through teach‐back responses and goal setting/compliance, and responses to a process evaluation survey at the end of the study. Sociodemographic characteristics were collected only at baseline.

### 2.6 Anthropometry

Study RDs measured the participants' height, weight, and WC using standardized procedures. Height (cm) was measured to the nearest 0.1 cm using a portable stadiometer (Perspective Enterprises, Portage); weight was measured to the nearest 100 g using an Omron Scale with participants wearing light indoor clothing and no shoes. Body mass index (BMI) [(weight (kg)/height (m)^2^] was calculated. Waist circumference was measured using a tape measure snugly fitted around each participant's waist at the level of the iliac crest and measuring the circumference after expiration.

### 2.7 Dietary intake

The 14‐item Mediterranean diet survey was used to assess compliance with the MedDiet reporting on habitual consumption of foods/beverages. Response categories are 1 or 0 (total range 0–14) representing participants' adherence to each food category in predetermined frequencies, including servings of fruit and vegetables, sweet desserts, breads/starches (refined vs. whole grains), ocean fish, and beverages. A higher score represents higher adherence to the MedDiet.^43^, ^44^

### 2.8 Physical activity

Daily steps were measured using a Fitbit that the participants were instructed to wear for seven consecutive days; the device can be clipped to the waistband or a front pocket and measure motion through an internal accelerometer aggregating into data as daily step count or time (minutes) spent in PA. A minimum of 3 days, including one weekend day of recorded data, was accepted for analysis.

### 2.9 Post‐traumatic stress disorder

Post‐traumatic stress disorder symptoms were measured using PCL‐5, a 17‐item self‐report validated measure of current (past month) PTSD symptom severity based on the DSM‐5 criteria. Participants were asked to rate problems they were bothered by in the past month “in relation to 9/11” on a scale of 1 = not at all to 5 = extremely (range 17–85). A minimum of 5 points suggests mild response to treatment; 5–10 points moderate, reliable change (e.g., change not due to chance); and a minimum of 10 points represents clinically meaningful change (for example, transition toward “symptoms closer to non‐PTSD”; 10–20 points clinically significant change “no‐PTSD”.^45^

### 2.10 Blood biomarkers

A total of up to 60 mL of blood was collected at baseline, post‐intervention, and follow‐up. Small aliquots of participant plasma samples were frozen and stored at −80°C at the WTC research facility located at Stony Brook University Hospital until all samples from the same group were collected and could be analyzed on the same day. Biomarkers included a detailed lipid panel [oxidized low‐density lipoprotein (oxLDL), calcLDL, high‐density lipoprotein, triglycerides], omega‐3 and omega‐6, Hemoglobin A1c (HbA1c), and high sensitivity C‐reactive protein; 10 inflammatory cytokines (IFNɣ, IL‐1β, IL‐4, IL‐5, IL‐6, IL‐8, IL‐10, IL‐12p70, IL‐22 and TNFα) were analyzed by Quanterix (CorPlex Cytokine profile).

### 2.11 Statistical analysis

To assess the study's primary aim, participant recruitment and retention were calculated. The number of participants who read the in‐app messages via their responses and compliance with goal setting were assessed. Descriptive statistics (frequencies/percent) of demographics at baseline and statistical comparisons of secondary outcomes (i.e., MedDiet score and blood biomarkers) by condition at three measurement times were calculated. Because the study was not powered for intervention efficacy, the sample size was limited and outcome variables did not follow a Gaussian distribution, between‐group comparisons were conducted for continuous outcomes using nonparametric Wilcoxon rank sum tests; pre‐post within‐group comparisons were conducted using Wilcoxon signed rank tests. It was also examined whether analytic choices might change the results using linear mixed models that were fitted for outcome measures with the inclusion of all three time points in adjusting for age and gender. While this method provides the flexibility to account for within‐subject correlation for repeated measures, it resulted in similar levels of significance as non‐parametric methods mentioned above; thus, the results in this study reflect the median values derived from nonparametric tests.

## 3 RESULTS

The average age of participants was 54 years (range: 42–64), 87% male, 58% Caucasian, 5% African American, 5% Asian/other/multiracial, and 32% refused to answer/unknown; more than one half of the participants had some college education and 20% had a bachelor's degree or grad school; close to 60% worked in law enforcement, 35% worked full time, and 40% were retired. The two groups were not significantly different in demographic characteristics (Table 2).

**TABLE 2:** Characteristics of participants in the WTC‐Nutrition study (*N* = 62).

Characteristics	Total *N* = 62	Intervention *N* = 31	Control *N* = 31	*p*‐value
Race (*n*, %)				
White or Caucasian	36 (58.06%)	16 (25.81%)	20 (32.26%)	0.740
Black or African‐American	3 (4.84%)	2 (3.23%)	1 (1.61%)	
Asian/Other/Multi‐racial	3 (4.84%)	2 (3.23%)	1 (1.61%)	
Gender: Male	54 (87.10%)	27 (43.55%)	27 (43.55%)	1.00
Age (mean; SD)	54.24 (5.80)	54.90 (6.14)	53.58 (5.46)	0.313
Age Range	42.00–64.00	42.00–64.00	43.00–64.00	
Education (*n*, %)				
High school graduate	12 (21.82%)	5 (9.09%)	7 (12.73%)	0.669
Associate, some college, tech school	30 (54.54%)	16 (27.28%)	15 (27.27%)	
Bachelor's Degree/Grad school	11 (20.00%)	8 (14.55%)	5 (9.09%)	

**FIGURE 1:**
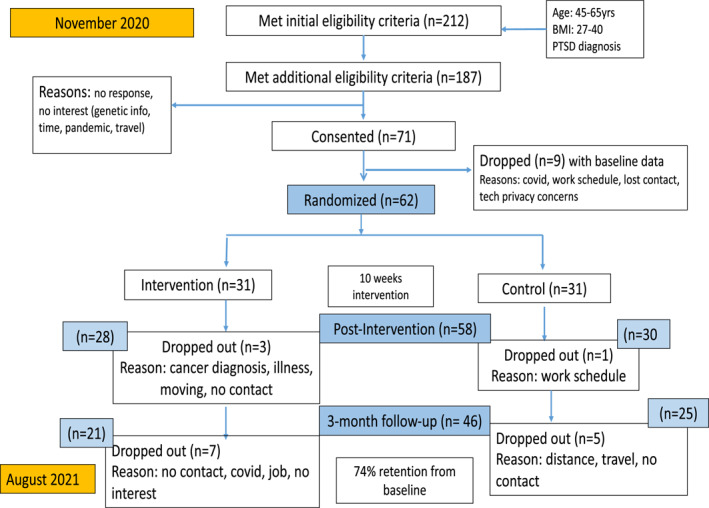
Recruitment, randomization, and data collection for the WTC‐Nutrition pilot randomized controlled trial.

Figure 1 shows a flowchart of recruitment, randomization, and data collection. A total of 212 WTC‐HP members met initial eligibility criteria within the past year based on their medical records; the main reasons for refusal to participate were either the COVID‐19 pandemic, lack of interest, time, or travel plans. Seventy‐one members consented to participate and completed baseline measures; nine participants dropped out prior to randomization due to concerns about COVID‐19, work schedule, or tech privacy, resulting in 62 randomized participants. A total of 58 (94%) participants completed the post‐intervention and 46 (74%) completed all follow‐up measurements. More than 85% of MedDiet participants watched the weekly videos and responded to teach back questions related to the video content and set a weekly goal; out of those who set goals, 41% fully completed and 21% partially completed their goals (made a few changes toward the set goal); 83% (20/24) reported being satisfied and very satisfied with the program, 96% (23/24) would participate again, and 100% (24/24) would recommend it to other WTC‐HP members. More than 90% were satisfied with the individual components (e.g., website, recipes, videos), 95% (23/24) were satisfied and very satisfied with the PA tips, while 92% (22/24) would like to have more cooking demonstrations in the future; 92% of MedDiet participants reported that receiving the can of olive oil at baseline helped increase intake and motivated them to purchase more.

Both groups experienced a significant drop in the median weight, BMI, and WC at post intervention and at follow‐up. Overall, at baseline the median (range) weight was 100.4 (73.7–135.2) kg, BMI 33.2 (27.5–41.8) kg/m^2^, and WC 109.2 (90.2–150.6) cm. At post‐intervention, there was a drop in median weight by [MedDiet −3% (−11%–7%) *p* = 0.0002 versus Control −1% (−13%–4%), *p* = 0.008] and at follow‐up by [MedDiet −2% (−14%–12%), *p* = 0.07 versus Control −2% (−20%–3%), *p* = 0.006)]. Overall, there was a reduction in BMI at post‐intervention by −0.68 (−4.61–2.09) kg/m^2^, *p* < 0.0001 and at follow‐up by −0.60 (−6.91–3.59) kg/m^2^, *p* = 0.0009). There was also a reduction in WC in both groups (*p* < 0.0001) by −3.1 (−33.0–3.1) cm and by −4.4 (−38.1–4.6) cm at post‐intervention and follow‐up (Table 3).

**TABLE 3:** Changes in weight, body mass index, and waist circumference (WC) among participants in the WTC‐Nutrition study (*N* = 62).

Outcome variables	Total *N* = 62	Intervention *N* = 31	Control *N* = 31	Between group *p*‐value
	Median (min, max) *p*‐value	Media (min, max) *p*‐value^a^	Median (min, max) *p*‐value^a^	
Weight (kg)				
Baseline	100.42 (72.66–135.17)	98.88 (80.10–135.17)	100.65 (72.66–130.09)	0.905
Median % weight change (baseline to post)	−2% (−13%–7%) ***p* < 0.0001**	−3% (−11%–7%) ***p* = 0.0002**	−1% (−13%–4%) ***p* = 0.008**	0.222
Median % weight change (baseline to 3‐month follow up)	−2% (−2%–12%) ***p* = 0.0008**	−2% (−14%–12%) ***p* = 0.07**	−2% (−20%–3%) ***p* = 0.006**	0.982
BMI (kg/m^2^)				
Baseline	33.20 (27.50–41.75)	33.61 (28.32–40.53)	32.75 (27.50–41.75)	0.387
Difference baseline to post‐intervention	−0.68 (−4.61–2.09) ***p* < 0.0001**	−0.82 (−3.78–2.09) ***p* = 0.0001**	−0.49 (−4.61–1.34) ***p* = 0.010**	0.173
Difference baseline to 3‐month follow up	−0.60 (−6.91–3.59) ***p* = 0.0009**	−0.59 (−4.63–3.59) *p* = 0.06	−0.75 (−6.91–1.35) ***p* = 0.007**	0.982
Waist circumference (cm)^b^				
Baseline	109.22 (90.17–150.62)	109.22 (90.17–150.62)	109.6 (94.61–142.24)	0.897
Difference baseline to post‐intervention	−3.81 (−33.00–3.30) ***p* < 0.0001**	−4.45 (−24.76–1.27) ***p* < 0.0001**	−2.54 (−33.02–3.30) **<0.0001**	0.168
Difference baseline to 3‐month follow up	−4.45 (−38.10–4.57) ***p* < 0.0001**	−6.35 (−24.13–1.90) ***p* < 0.0001**	−4.19 (−38.10–4.57) **<0.0001**	0.466

*Note*: Inclusion criteria were BMI ≥27 to ≤40. The bolded values indicate statistically significant improvement in the values where the *p*‐value is lower than 0.05.

Abbreviation: BMI, Body mass index (kg/m^2^).

^a^ *p*‐values indicate within group changes.

^b^ Low risk: Women: <80 cm (31.5 in); Men: <94 cm (37 in).

Both groups experienced an increase in the MedDiet score; at baseline, the median MedDiet score was 6.0 (1.0–11.0) and increased by 4 points (−1.0–8.0, *p* < 0.0001) and 3 points (−2.0–7.0, *p* < 0.0001) at post‐intervention and follow‐up, respectively. Among all participants, the median PCL score at baseline was 52.00 (25.00–74.00) and decreased by −13 points (−37.00–11.00), *p* < 0.0001 and by −8 points (−61.00–14.00) *p* < 0.0001 at post‐intervention and follow‐up, respectively (Table 4).

**TABLE 4:** Changes in the Mediterranean diet score and PCL score among participants in the WTC‐Nutrition study (*N* = 62).

Outcome variables	Total *N* = 62	Intervention *N* = 31	Control *N* = 31	Between group *p*‐value
	Median (min, max) *p*‐value^b^	Median (min, max) *p*‐value^b^	Median (min, max) *p*‐value^b^	
Mediterranean diet score^a^				
Baseline	6.00 (1.00–11.00)	6.00 (1.00–11.00)	6.00 (1.00–10.00)	
Difference baseline to post‐intervention	4.00 (−1.00–8.00) ***p* < 0.0001**	4.00 (−1.00–8.00) ***p* < 0.0001**	3.00 (−1.00–7.00) ***p* < 0.0001**	0.099
Difference baseline to 3‐month follow up	3.00 (−2.00–7.00) ***p* < 0.0001**	4.00 (−1.00–6.00) ***p* < 0.0001**	3.00 (−2.00–7.00) ***p* < 0.0001**	0.986
PCL score^c^				
Baseline	52.00 (25.00–74.00)	52.00 (31.00–71.00)	50.00 (25.00–74.00)	0.927
Difference baseline to post‐intervention	−13.00 (−37.00–11.00) ***p* < 0.0001**	−11.50 (−37.00–6.00) ***p* < 0.0001**	−13.00 (−30.00–11.00) ***p* < 0.0001**	1.00
Difference baseline to 3‐month follow up	−8.00 (−61.00–14.00) ***p* < 0.0001**	−6.50 (−61.00–14.00) ***p* = 0.001**	−9.50 (−25.00–10.00) ***p* < 0.0001**	0.741

*Note*: The bolded values indicate statistically significant improvement in the values where the *p*‐value is lower than 0.05.

^a^ Mediterranean Diet Score Range: 1–14 (higher score indicates more adherence to the Mediterranean Diet (response Yes = 1; No = 0).

^b^ *p*‐values indicate within group changes.

^c^ Possible PCL Score Range: 17–85 (higher score indicates more severe PTSD symptoms).

Table 5 shows changes in oxLDL and other lipid biomarkers; oxLDL was reduced in both groups without significant group differences. For the total sample, the baseline median (range) oxLDL was 48.5 (19.0–570.0) U/L and was reduced by −12.50 (−352.0–82.0) U/L, *p* < 0.0001 at post‐intervention and by −24.00 (−391.00–130.00) U/L, *p* < 0.0001 at follow‐up. Only the MedDiet group experienced a significant drop in median total cholesterol at post‐intervention; at the baseline, the median total cholesterol was 200.00 (100.00–293.00) mg/dL and was reduced by −8.5 (−88.00–38.00) mg/dL, *p* = 0.035.

**TABLE 5:** Changes in blood biomarkers of hsCRP and HA1c, lipids, and fatty acid ratio among participants in the WTC‐Nutrition study (*N* = 62).

Outcome Variables	Total *N* = 62	Intervention *N* = 31	Control *N* = 31	Between group *p*‐value
	Median (min, max) *p*‐value^a^	Median (min, max) Within group *p*‐value^a^	Median (min, max) Within group *p*‐value^a^	
Oxidized LDL (U/L)				
Baseline	48.50 (19.00–570.00)	40.00 (19.0–570.0	68.00 (21.00–470.00)	0.119
Difference baseline to post‐intervention	−12.50 (−352.0–82.00) ***p* < 0.0001**	−5.00 (−352.0–31.00) **0.027**	−16.00 (−286.0–82.00) **<0.0001**	0.117
Difference baseline to 3‐month follow up	−24.00 (−391.00–130.00) ***p* < 0.0001**	−20.00 (−391.00–130.0) **0.002**	−26.00 (−287.00–12.00) **<0.0001**	0.638
hs‐CRP (mg/L)				
Baseline	1.80 (0.30–9.40)	1.70 (0.3–9.4)	1.90 (0.70–7.0)	0.680
Difference baseline to post‐intervention	0.00 (−2.60–3.00) *p* = 0.76	−0.30 (−2.60–3.00) 0.255	0.25 (−2.60–2.60) 0.167	0.101
Difference baseline to 3‐month follow up	0.20 (−4.30–3.90) *p* = 0.471	−0.18 (−4.30–2.20) 0.238	0.25 (−1.90–3.90) **0.059**	**0.025**
Hemoglobin A1c (%)				
Baseline	5.70 (4.80–11.70)	5.90 (4.80–7.70)	5.60 (5.00–11.70)	0.158
Difference baseline to post‐intervention	0.00 (−1.20–0.60) *p* = 0.069	−0.10 (−1.00–0.30) **0.009**	0.00 (−1.20–0.60) 0.757	**0.019**
Difference baseline to 3‐month follow up	0.05 (−1.20–2.70) *p* = 0.518	0.10 (−1.20–1.20) 0.790	0.00 (−0.90–2.70) 0.647	0.657
Total cholesterol (mg/dL)				
Baseline	200.50 (100.0–293.0)	200.00 (100.00–293.00)	201.00 (138.00–270.00)	0.811
Difference baseline to post	−9.00 (−93.00–51.00) ***p* = 0.007**	−8.50 (−88.00–38.00) ***p* = 0.035**	−9.00 (−93.00–51.00) *p* = 0.092	0.907
Difference baseline to 3‐month follow up	−13.9 (−110.0–73.0) ***p* = 0.031**	−8.00 (−110.0–55.00) *p* = 0.107	−16.00 (−102.0–73.00) *p* = 0.142	0.683
CHOL/HDL ratio				
Baseline	4.05 (2.20–10.40)	4.00 (2.20–10.40)	4.20 (2.80–7.60)	0.844
Difference baseline to post	−0.10 (−6.40–1.50) *p* = 0.132	−0.15 (−6.40–0.80) *p* = 0.231	−0.10 (−3.10–1.50) *p* = 0.353	0.803
Difference baseline to 3‐month follow up	−0.25 (−6.60–1.40) ***p* = 0.015**	−0.40 (−6.60–1.00) ***p* = 0.032**	−0.20 (−2.40–1.40) *p* = 0.170	0.396
Triglycerides				
Baseline	129.50 (55.00–702.00)	130.00 (60.00–702.00)	129.00 (55.00–357.00)	0.741
Difference baseline to post	−19.00 (−569.0–124.00) **p = 0.012**	−16.50 (−569.0–124.00) *p* = 0.212	−21.50 (−170.0–64.00) *p* = **0.022**	0.768
Difference baseline to 3‐month follow up	−12.00 (−572.0–97.00) *p* = 0.062	−15.00 (−572.0–97.00) *p* = 0.154	−3.00 (−145.0–91.00) *p* = 0.184	0.825
ω6/ω3 ratio				
Baseline	14.38 (6.85–25.24)	14.57 (6.85–22.86)	14.32 (7.88–25.24)	0.994
Difference baseline to post	−1.69 (−8.84–6.72) ***p* = 0.003**	−2.71 (−8.84–6.72) ***p* = 0.002**	−0.29 (−7.53–4.56) *p* = 0.307	**0.029**
Difference baseline to 3‐month follow up	−0.97 (−7.88–12.22) *p* = 0.263	−1.52 (−7.88–5.07) *p* = 0.092	0.03 (−3.67–12.22) *p* = 0.990	0.152

*Note*: Normal range: Oxidized LDL (U/L) < 60 U/L. hs‐CRP (mg/L): 1.0–3.0 mg/L. Hemoglobin A1c (%): prediabetes range 5.7%–6.4% based on the specific lab. Total Cholesterol (mg/dL): <200 mg/dL. CHOL/HDL ratio: ≤3.5 indicate low‐risk for CVD. Triglycerides: <150 mg/dL. ω6/ω3: Lower ratio indicates a less inflammatory state (desirable). The bolded values indicate statistically significant improvement in the values where the *p*‐value is lower than 0.05.

^a^ *p*‐values indicate within group changes.

**FIGURE 2:**
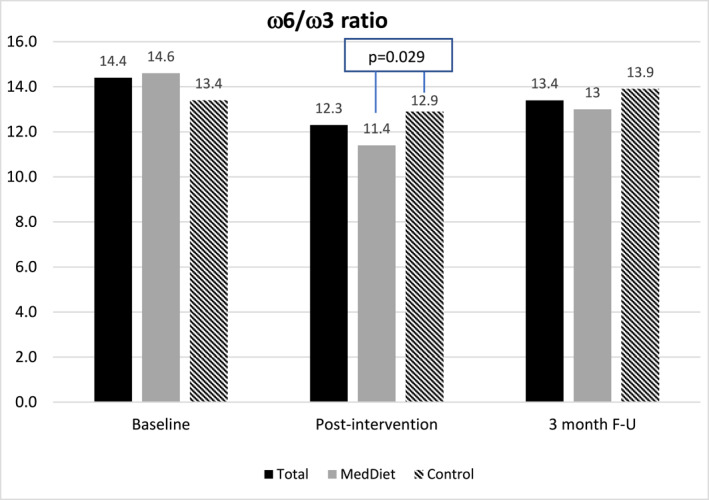
Serum omega 6‐to‐omega 3 ratio significant treatment effect between Mediterranean Diet and Control groups at post‐intervention, *p* = 0.029.

There were treatment differences in HbA1c, ω6/ω3, and hsCRP; the MedDiet group experienced reduction in HbA1c [MedDiet: −0.10% (−1.00–0.30), *p* = 0.009 versus Control: 0.00% (−1.20–0.60); *p* = 0.757; between group diff. *p* = 0.019] and in ω6/ω3 [MedDiet: −2.7 (−8.84–6.72, *p* = 0.002) versus Control: −0.29 (−7.53–4.56; *p* = 0.307); between group *p* = 0.029) at post‐intervention (Figure 2).

**FIGURE 3:**
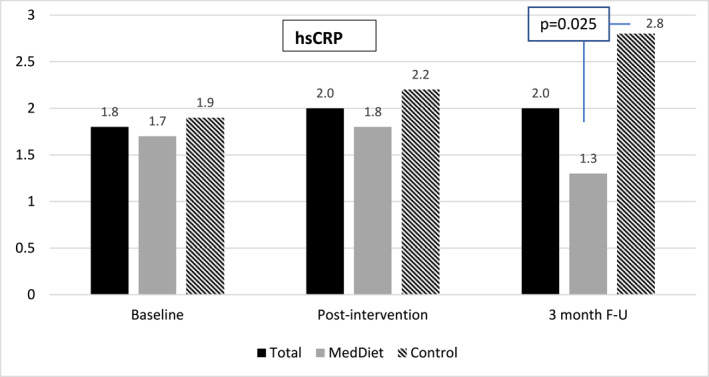
Serum hsCRP significant treatment effect between the Mediterranean Diet and Control groups at 3‐month follow‐up, *p* = 0.025.

While hsCRP did not significantly decrease in the total sample, at follow‐up there was a significant treatment difference (*p* = 0.025) because the MedDiet group experienced a decrease of −0.18 mg/L, while the control group experienced an increase of 0.25 mg/L (Figure 3). On average, the participants recorded about 7300 steps per day without significant changes within or between groups over the study period (data not shown). There were no significant within or between group changes in inflammatory cytokines thus results were not presented here.

## 4 DISCUSSION

To address the perceived gap in lifestyle interventions among WTC responders who suffer from both somatic and mental issues, the WTC‐Nutrition pilot intervention was conducted in a group of WTC responders with overweight and obesity and chronic PTSD. The study met the primary outcome of feasibility and acceptability, having successfully recruited and retained a sample of 62 participants. The majority of MedDiet participants expressed a high degree of satisfaction with the program and the weekly education materials and unanimously reported that they would recommend it to other clinic patients.

In interpreting the secondary outcomes of this study, it is important to consider that the control group (usual care) consisted of nutrition counseling with healthful dietary recommendations. While intervention effectiveness was not the primary aim of the study,^46^ intent‐to‐treat analyses indicated that both groups showed significant and sustained improvements in body weight, WC, BMI, oxLDL and total cholesterol, while the MedDiet group, compared to control, had additional improvements in HA1c, ω6/ω3, and hsCRP at either post‐intervention or at follow‐up. Despite the lack of significant change in hsCRP in either group, the group difference that was driven by a decrease among the MedDiet group and an increase in the control group may provide support for the anti‐inflammatory properties of the MedDiet.^26^ Additionally, both groups experienced improvements in their PTSD symptoms.

In this 10‐week pilot intervention the participants experienced an overall median weight reduction of 2%; while it was lower than the clinical significance threshold of >5%, the results were similar to a large study among 60,000 U.S veterans with overweight/obesity and PTSD; in the study reporting 6‐month outcomes of the VA MOVE!, a multidisciplinary weight management program, the overall mean percent change in weight was −1.9.^47^ In contrast, a weight loss counseling study by Haas and colleagues that was delivered through a mobile phone app among 43 adults with overweight and obesity found median weight loss of −4.6%, reduction in BMI of −1.4, and WC reduction of −3.5 cm (1.4 inches).^48^ In the present WTC‐Nutrition study the MedDiet group experienced a larger drop in the WC of −1.75 inches at post intervention and −2.5 inches and at 3‐month follow up. While both studies used a mobile technology, the study by Hass used a mobile app with tailored counseling and feedback.^48^ Other notable differences were that the Haas study included participants with lower BMI (26–33 kg/m^2^) and without mental health issues. Nevertheless, considering the consistent findings that individuals with PTSD participating in weight loss programs have lower rates of success,^49^ the anthropometric improvements of this WTC‐Nutrition pilot study were encouraging.

Favorable changes in the self‐reported MedDiet score from a median of 6 to 10 (out of 14) indicate compliance with the recommended dietary changes. Furthermore, the dietary improvement coupled with the reduction of serum omega‐6/omega‐3 ratio, especially in the MedDiet group, further confirmed the increase in the anti‐inflammatory diet. While both omega 3 and 6 are essential fatty acids, they have different effects on disease risk.^50^ Based on research to date, within an overall healthy diet, a recommended ratio of about 4:1 has shown to have a favorable impact on inflammation and health promotion.^51^ At baseline, study participants had a median ratio of 14.4 which was consistent with the average Western diet.^52^ The results of this study suggest that a multidomain lifestyle intervention with a focus on the MedDiet can improve the omega‐6/omega‐3 ratio in patients with overweight and obesity and comorbid PTSD.

While improvement in PTSD symptoms was not an aim of this study, it is noteworthy that both groups experienced a significant reduction in PCL scores. A 13.0‐point reduction at post‐intervention was within the clinically meaningful change, indicating a transition toward non‐PTSD. Various potential factors could have contributed to these changes, including but not limited to nutrition counseling serving as psychotherapy due to individualized attention and promotion of self‐care; or it could be due to positive reduction in weight and various inflammatory and oxidative biomarkers, supporting prior findings on the interconnection of weight, atherogenic lipid profile, and systemic inflammation, all risk factors for PTSD.^14^, ^15^, ^53^, ^54^ Trials among military and veteran populations with PTSD have shown that first‐line psychotherapy approaches have high dropout rates and many patients remain highly symptomatic post treatment.^55^ Having high participant retention, compliance, and satisfaction in this study, nutrition programming might be an indirect method for providing some relief for PTSD symptoms. Emerging evidence of the association between combat‐related PTSD and higher levels of pro‐inflammatory cytokines further supports lifestyle interventions to address both the psychiatric and somatic aspects of PTSD.^56^, ^57^

While a larger study is needed to confirm the current findings, the significant improvements in oxLDL are promising, as oxLDL plays a primary role in MetS‐related diseases leading to numerous pro‐inflammatory and pre‐carcinogenic effects. This study, therefore, adds to a growing body of research examining the effects of diet, especially MedDiet, on oxLDL levels.^58^, ^59^, ^60^, ^61^ The reduction of total cholesterol among the MedDiet group is in agreement with findings from other randomized studies.^61^, ^62^ Additionally, numerous studies have indicated the protective effect of the MedDiet on the cardiovascular and vascular health.^25^, ^28^, ^63^, ^64^ A study comparing the diets of individuals with acute ischemic stroke with healthy controls indicated a higher prevalence of stroke and worse clinical profile among those with low adherence to MedDiet.^65^

There were strengths and limitations to this study. In addition to limitations inherent to every pilot study (e.g., limited power), this study included WTC responders, a predominantly white male population exposed to a unique circumstance who also work in a small number of response‐focused occupations; thus, the findings may not be generalizable. In addition, study participants may be more eager to make lifestyle changes, thus positively contributing to the study's success. The relatively low rate of participants who achieved their personal goals will need further investigation in future studies.

Yet, this study also had several strengths. To the authors' knowledge, this was the first nutrition intervention implemented among the WTC responders with overweight and obesity and diagnosed PTSD and has the potential to serve as a guide in augmenting treatment activities among the WTC responders. The primary study aim was successfully met by demonstrating successful recruitment in the WTC‐HP clinics, engaging the participants throughout the intervention, and retaining them across all three assessments; however, the summer season may have partially contributed to slightly lower participation in follow‐up measurements. Using a multimodal approach to intervention delivery, mainly the Internet and mobile phones and augmented by individual counseling, might have significantly contributed to the improvement of eating behaviors and health outcomes as it has been shown in other studies.^66^, ^67^ Most MedDiet participants responded to the weekly educational sessions with high compliance in answering the teach back questions. The study utilized objective measures such as serum biomarkers and validated measures to assess dietary intake.

## 5 CONCLUSION

The WTC‐Nutrition intervention implemented MedDiet, that has been extensively researched and established as a heart healthy diet shown to decrease systemic inflammation and risk for cardiovascular disease. The intervention was mostly delivered digitally that has allowed for enhanced communication frequency and concomitant increased intervention dose, as well as convenience among those with shift‐related occupations. Additionally, since the study was conducted during a period marked by efforts to reduce exposure to COVID‐19, this delivery method allowed the study to continue given the required restrictions. Despite the remote delivery, the results were consistent with previous studies showing that nutritional interventions can effectively reduce metabolic risk factors.^26^, ^28^ Furthermore, when accompanied by regular contact and individual goal setting, as in this study, nutritional interventions can potentially improve weight and mental health in participants with comorbid overweight and obesity and mental health conditions. In summary, the study successfully met its aims and provided valuable lessons that can be used to guide the development of a full‐scale trial, while components of the intervention can be implemented in clinics serving populations with comorbid physical and mental health issues.

## AUTHOR CONTRIBUTIONS

Chrisa Arcan conceived the study and led the development of the manuscript; Wei Hou analyzed data; Kathryn Hoffman and Amanda Reichardt served as study dietitians, developed the study materials, and assisted in study coordination; Xiaohua Yang advised and coordinated blood collection, specimen storage and quality control of blood analysis results; Sean A. P. Clouston advised on study design; Benjamin Luft directed all study activities within the Clinic operations and insured smooth study completion; all authors critically reviewed the manuscript and had final approval of the submitted manuscript.

## CONFLICT OF INTEREST STATEMENT

The authors declare no conflicts of interest.

## CLINICAL TRIAL REGISTRATION

www.clinicaltrials.gov: NCT05138198.

## References

[1] Smith EC , Holmes L , Burkle FM . The physical and mental health challenges experienced by 9/11 first responders and recovery workers: a review of the literature. Prehosp Disaster Med. 2019;34(6):625‐631. 10.1017/S1049023X19004989 31625489

[2] Clouston S , Pietrzak RH , Kotov R , et al. Traumatic exposures, posttraumatic stress disorder, and cognitive functioning in World Trade Center responders. Alzheimers Dement (N Y). 2017;3(4):593‐602. 10.1016/j.trci.2017.09.001 29201993 PMC5700827

[3] Clouston SA , Kotov R , Pietrzak RH , et al. Cognitive impairment among World Trade Center responders: long‐term implications of re‐experiencing the 9/11 terrorist attacks. Alzheimers Dement (Amst). 2016;4(1):67‐75. 10.1016/j.dadm.2016.08.001 27626057 PMC5011166

[4] Waszczuk MA , Li K , Ruggero CJ , Clouston SAP , Luft BJ , Kotov R . Maladaptive personality traits and 10‐year course of psychiatric and medical symptoms and functional impairment following trauma. Ann Behav Med. 2018;52(8):697‐712. 10.1093/abm/kax030 30010707

[5] Moline JM , McLaughlin MA , Sawit ST , et al. The prevalence of metabolic syndrome among law enforcement officers who responded to the 9/11 World Trade Center attacks. Am J Ind Med. 9AD. 2016;59(9):752‐760. 10.1002/ajim.22649 27582477

[6] World Health Organization . The ICD‐10 Classification of Mental and Behavioural Disorders: Clinical Descriptions and Diagnostic Guidelines; 1992. Published online.

[7] Bromet EJ , Hobbs MJ , Clouston SA , Gonzalez A , Kotov R , Luft BJ . DSM‐IV post‐traumatic stress disorder among World Trade Center responders 11–13 years after the disaster of 11 September 2001 (9/11). Psychol Med. 2016;46(4):771‐783. 10.1017/s0033291715002184 26603700 PMC4754831

[8] Wolf EJ , Miller DR , Logue MW , et al. Contributions of polygenic risk for obesity to PTSD‐related metabolic syndrome and cortical thickness. Brain Behav Immun. 2017;65:328‐336. 10.1016/j.bbi.2017.06.001 28579519 PMC5537007

[9] Wolf EJ , Bovin MJ , Green JD , et al. Longitudinal associations between post‐traumatic stress disorder and metabolic syndrome severity. Psychol Med. 2016;46(10):2215‐2226. 10.1017/S0033291716000817 27087657 PMC4925183

[10] Rosenbaum S , Stubbs B , Ward PB , Steel Z , Lederman O , Vancampfort D . The prevalence and risk of metabolic syndrome and its components among people with posttraumatic stress disorder: a systematic review and meta‐analysis. Metabolism. 2015;64(8):926‐933. 10.1016/j.metabol.2015.04.009 25982700

[11] Hoerster KD , Lai Z , Goodrich DE , et al. Weight loss after participation in a national VA weight management program among veterans with or without PTSD. Psychiatr Serv. 2014;65(11):1385‐1388. 10.1176/appi.ps.201300404 25123784

[12] Farr OM , Sloan DM , Keane TM , Mantzoros CS . Stress‐ and PTSD‐associated obesity and metabolic dysfunction: a growing problem requiring further research and novel treatments. Metabolism. 2014;63(12):1463‐1468. 10.1016/j.metabol.2014.08.009 25267015 PMC4459590

[13] van den Berk‐Clark C , Secrest S , Walls J , et al. Association between posttraumatic stress disorder and lack of exercise, poor diet, obesity, and co‐occurring smoking: a systematic review and meta‐analysis. Health Psychol. 2018;37(5):407‐416. 10.1037/hea0000593 29698016 PMC5922789

[14] Gill J , Luckenbaugh D , Charney D , Vythilingam M . Sustained elevation of serum interleukin‐6 and relative insensitivity to hydrocortisone differentiates posttraumatic stress disorder with and without depression. Biol Psychiatry. 2010;68(11):999‐1006. 10.1016/j.biopsych.2010.07.033 20951370

[15] Gola H , Engler H , Sommershof A , et al. Posttraumatic stress disorder is associated with an enhanced spontaneous production of pro‐inflammatory cytokines by peripheral blood mononuclear cells. BMC Psychiatr. 2013;13(1):40. 10.1186/1471-244X-13-40 PMC3574862 23360282

[16] Speer K , Upton D , Semple S , McKune A . Systemic low‐grade inflammation in post‐traumatic stress disorder: a systematic review. J Inflamm Res. 2018;11:111‐121. 10.2147/JIR.S155903 29606885 PMC5868606

[17] Brite J , Friedman S , de la Hoz RE , Reibman J , Cone J . Mental health, long‐term medication adherence, and the control of asthma symptoms among persons exposed to the WTC 9/11 disaster. J Asthma. 11AD. 2020;57(11):1253‐1262. 10.1080/02770903.2019.1672722 PMC7594532 31550944

[18] Ngandu T , Lehtisalo J , Solomon A , et al. A 2 year multidomain intervention of diet, exercise, cognitive training, and vascular risk monitoring versus control to prevent cognitive decline in at‐risk elderly people (FINGER): a randomised controlled trial. Lancet. 2015;385(9984):2255‐2263. 10.1016/S0140-6736(15)60461-5 25771249

[19] Theal R , Tay VXP , Hickman IJ . Conflicting relationship between dietary intake and metabolic health in PTSD: a systematic review. Nutr Res. 6AD. 2018;54:12‐22. 10.1016/j.nutres.2018.03.002 29914663

[20] Menzel J , Jabakhanji A , Biemann R , Mai K , Abraham K , Weikert C . Systematic review and meta‐analysis of the associations of vegan and vegetarian diets with inflammatory biomarkers. Sci Rep. 2020;10(1):21736. 10.1038/s41598-020-78426-8 33303765 PMC7730154

[21] Browne J , Morey MC , Beckham JC , et al. Diet quality and exercise in older veterans with PTSD: a pilot study. Transl Behav Med. 2021;11(12):2116‐2122. 10.1093/tbm/ibab116 34487181 PMC8846334

[22] Davis C , Bryan J , Hodgson J , Murphy K . Definition of the Mediterranean diet: a literature review. Nutrients. 2015;7(11):9139‐9153. 10.3390/nu7115459 26556369 PMC4663587

[23] Dominguez LJ , Barbagallo M . Nutritional prevention of cognitive decline and dementia. Acta Biomed. 2018;89(2):276‐290. 10.23750/abm.v89i2.7401 29957766 PMC6179018

[24] Shapira N . The metabolic concept of meal sequence vs. Satiety: glycemic and oxidative responses with reference to inflammation risk, protective principles and mediterranean diet. Nutrients. 2019;11(10):2373. 10.3390/nu11102373 31590352 PMC6835480

[25] Tuttolomondo A , Simonetta I , Daidone M , Mogavero A , Ortello A , Pinto A . Metabolic and vascular effect of the Mediterranean diet. Int J Mol Sci. 2019;20(19):4716. 10.3390/ijms20194716 31547615 PMC6801699

[26] Razquin C , Martinez‐Gonzalez MA . A traditional Mediterranean diet effectively reduces inflammation and improves cardiovascular health. Nutrients. 2019;11(8):1842. 10.3390/nu11081842 31395816 PMC6723673

[27] van den Brink AC , Brouwer‐Brolsma EM , Berendsen AAM , van de Rest O . The Mediterranean, dietary approaches to stop hypertension (DASH), and Mediterranean‐DASH intervention for neurodegenerative delay (MIND) diets are associated with less cognitive decline and a lower risk of Alzheimer’s disease‐A review. Adv Nutr. 2019;10(6):1040‐1065. 10.1093/advances/nmz054 31209456 PMC6855954

[28] Papadaki A , Nolen‐Doerr E , Mantzoros CS . The effect of the Mediterranean diet on metabolic health: a systematic review and meta‐analysis of controlled trials in adults. Nutrients. 2020;12(11):3342. 10.3390/nu12113342 33143083 PMC7692768

[29] Tosti V , Bertozzi B , Fontana L . Health benefits of the Mediterranean diet: metabolic and molecular mechanisms. J Gerontol A Biol Sci Med Sci. 2018;73(3):318‐326. 10.1093/gerona/glx227 29244059 PMC7190876

[30] Hutchesson MJ , Rollo ME , Krukowski R , et al. eHealth interventions for the prevention and treatment of overweight and obesity in adults: a systematic review with meta‐analysis. Obes Rev. 2015;16(5):376‐392. 10.1111/obr.12268 25753009

[31] Gong Y , Jiang X , Chen X , et al. Effectiveness of mHealth diet interventions in cancer survivors: a systematic review and meta‐analysis of randomized controlled trials. Asia Pac J Oncol Nurs. 2023;10(3):100196. 10.1016/j.apjon.2023.100196 37124242 PMC10140457

[32] Ferguson JA , Daley AJ , Parretti HM . Behavioural weight management interventions for postnatal women: a systematic review of systematic reviews of randomized controlled trials. Obes Rev. 2019;20(6):829‐841. 10.1111/obr.12834 30941875

[33] Coughlin JW , Martin LM , Henderson J , et al. Feasibility and acceptability of a remotely‐delivered behavioural health coaching intervention to limit gestational weight gain. Obesity Science & Practice. 2020;6(5):484‐493. 10.1002/osp4.438 33082990 PMC7556432

[34] Fulkerson JA , Rydell S , Kubik MY , et al. Healthy Home Offerings via the Mealtime Environment (HOME): feasibility, acceptability, and outcomes of a pilot study. Obesity. 2010;18(Suppl 1):S69‐S74. 10.1038/oby.2009.434 20107464 PMC3070470

[35] Hopkins LC , Fristad M , Goodway JD , et al. Feasibility and acceptability of technology‐based caregiver engagement strategies delivered in a summertime childhood obesity prevention intervention: results from an internal pilot of the Camp NERF (Nutrition, Education, Recreation, and Fitness) study. Pilot Feasibility Stud. 2018;4(1):153. 10.1186/s40814-018-0340-2 30275966 PMC6158889

[36] Clouston SAP , Guralnik JM , Kotov R , Bromet EJ , Luft BJ . Functional limitations among responders to the World trade center attacks 14 Years after the disaster: implications of chronic posttraumatic stress disorder. J Trauma Stress. 10AD. 2017;30(5):443‐452. 10.1002/jts.22219 PMC5679479 29024005

[37] Fitbit . https://www.fitbit.com/home

[38] Bandura A . Health promotion by social cognitive means. Health Educ Behav official Publ Soc Public Health Educ. 2004;31(2):143‐164. 10.1177/1090198104263660 15090118

[39] Sotos‐Prieto M , Cash SB , Christophi CA , et al. Rationale and design of feeding America’s bravest: Mediterranean diet‐based intervention to change firefighters’ eating habits and improve cardiovascular risk profiles. Contemp Clin Trials. 2017;61:101‐107. 10.1016/j.cct.2017.07.010 28710052

[40] Elliot DL , Goldberg L , Duncan TE , et al. The PHLAME firefighters’ study: feasibility and findings. Am J Health Behav. 2004;28(1):13‐23. 10.5993/ajhb.28.1.2 14977155

[41] McCarroll ML , Armbruster S , Pohle‐Krauza RJ , et al. Feasibility of a lifestyle intervention for overweight/obese endometrial and breast cancer survivors using an interactive mobile application. Gynecol Oncol. 2015;137(3):508‐515. 10.1016/j.ygyno.2014.12.025 25681782

[42] Connolly Schoonen J Losing Weight Permanently with the Bull’s‐Eye Food Guide; 2004.

[43] García‐Conesa MT , Philippou E , Pafilas C , et al. Exploring the validity of the 14‐item Mediterranean diet adherence screener (MEDAS): a cross‐national study in seven European countries around the Mediterranean region. Nutrients. 2020;12(10):2960. 10.3390/nu12102960 32992649 PMC7601687

[44] Papadaki A , Johnson L , Toumpakari Z , et al. Validation of the English version of the 14‐item Mediterranean diet adherence screener of the PREDIMED study, in people at high cardiovascular risk in the UK. Nutrients. 2018;10(2):138. 10.3390/nu10020138 29382082 PMC5852714

[45] US Department of Veteran Affairs . PTSD Checklist for DSM‐5 (PCL‐5); 2022. Published. https://www.ptsd.va.gov/professional/assessment/adult‐sr/ptsd‐checklist.asp

[46] Abbott JH . The distinction between randomized clinical trials (RCTs) and preliminary feasibility and pilot studies: what they are and are not. J Orthop Sports Phys Ther. 2014;44(8):555‐558. 10.2519/jospt.2014.0110 25082389

[47] Batch BC , Goldstein K , Yancy WS , et al. Outcome by gender in the veterans health Administration motivating overweight/obese veterans everywhere weight management program. J Womens Health (Larchmt). 2018;27(1):32‐39. 10.1089/jwh.2016.6212 28731844 PMC5771533

[48] Haas K , Hayoz S , Maurer‐Wiesner S . Effectiveness and feasibility of a remote lifestyle intervention by dietitians for overweight and obese adults: pilot study. JMIR Mhealth Uhealth. 2019;7(4):e12289. 10.2196/12289 30973338 PMC6482396

[49] Klingaman EA , Hoerster KD , Aakre JM , Viverito KM , Medoff DR , Goldberg RW . Veterans with PTSD report more weight loss barriers than Veterans with no mental health disorders. Gen Hosp Psychiatry. 2016;39:1‐7. 10.1016/j.genhosppsych.2015.11.003 26719103

[50] Wu JHY , Micha R , Mozaffarian D . Dietary fats and cardiometabolic disease: mechanisms and effects on risk factors and outcomes. Nat Rev Cardiol. 10AD. 2019;16(10):581‐601. 10.1038/s41569-019-0206-1 31097791

[51] DiNicolantonio JJ , O’Keefe JH . Importance of maintaining a low omega‐6/omega‐3 ratio for reducing inflammation. Open Heart. 2018;5(2):e000946. 10.1136/openhrt-2018-000946 30564378 PMC6269634

[52] Simopoulos AP . The importance of the ratio of omega‐6/omega‐3 essential fatty acids. Biomed Pharmacother. 2002;56(8):365‐379. 10.1016/s0753-3322(02)00253-6 12442909

[53] Maes M , De Backer G , Suy E , Minner B . Increased plasma serine concentrations in depression. Neuropsychobiology. 1995;31(1):10‐15. 10.1159/000119166 7708176

[54] Vereecken CA , Todd J , Roberts C , Mulvihill C , Maes L . Television viewing behaviour and associations with food habits in different countries. Publ Health Nutr. 2006;9(2):244‐250. 10.1079/phn2005847 16571179

[55] Steenkamp MM , Litz BT , Hoge CW , Marmar CR . Psychotherapy for military‐related PTSD: a review of randomized clinical trials. JAMA. 2015;314(5):489‐500. 10.1001/jama.2015.8370 26241600

[56] Lindqvist D , Wolkowitz OM , Mellon S , et al. Proinflammatory milieu in combat‐related PTSD is independent of depression and early life stress. Brain, Behav Immun. 2014;42:81‐88. 10.1016/j.bbi.2014.06.003 24929195

[57] Bauer IE , Gálvez JF , Hamilton JE , et al. Lifestyle interventions targeting dietary habits and exercise in bipolar disorder: a systematic review. J Psychiatr Res. 2016;74:1‐7. 10.1016/j.jpsychires.2015.12.006 26724541 PMC4744495

[58] De Lorenzo A , Bernardini S , Gualtieri P , et al. Mediterranean meal versus Western meal effects on postprandial ox‐LDL, oxidative and inflammatory gene expression in healthy subjects: a randomized controlled trial for nutrigenomic approach in cardiometabolic risk. Acta Diabetol. 2017;54(2):141‐149. 10.1007/s00592-016-0917-2 27709360

[59] Martín‐Peláez S , Fito M , Castaner O . Mediterranean diet effects on type 2 diabetes prevention, disease progression, and related mechanisms. A Review. Nutrients. 2020;12(8):2236. 10.3390/nu12082236 32726990 PMC7468821

[60] Elkan AC , Sjöberg B , Kolsrud B , Ringertz B , Hafström I , Frostegård J . Gluten‐free vegan diet induces decreased LDL and oxidized LDL levels and raised atheroprotective natural antibodies against phosphorylcholine in patients with rheumatoid arthritis: a randomized study. Arthritis Res Ther. 2008;10(2):R34. 10.1186/ar2388 18348715 PMC2453753

[61] Hernáez Á , Castañer O , Goday A , et al. The Mediterranean Diet decreases LDL atherogenicity in high cardiovascular risk individuals: a randomized controlled trial. Mol Nutr Food Res. 2017;61(9):1601015. 10.1002/mnfr.201601015 28371298

[62] Estruch R , Martínez‐González MA , Corella D , et al. Effects of a Mediterranean‐style diet on cardiovascular risk factors: a randomized trial. Ann Intern Med. 2006;145(1):1‐11. 10.7326/0003-4819-145-1-200607040-00004 16818923

[63] Estruch R , Ros E , Salas‐Salvadó J , et al. Primary prevention of cardiovascular disease with a Mediterranean diet supplemented with extra‐virgin olive oil or nuts. N Engl J Med. 2018;378(25):e34. 10.1056/NEJMoa1800389 29897866

[64] Antoniazzi L , Arroyo‐Olivares R , Bittencourt MS , et al. Adherence to a Mediterranean diet, dyslipidemia and inflammation in familial hypercholesterolemia. Nutr Metabol Cardiovasc Dis. 2021;31(7):2014‐2022. 10.1016/j.numecd.2021.04.006 34039501

[65] Tuttolomondo A , Casuccio A , Buttà C , et al. Mediterranean Diet in patients with acute ischemic stroke: relationships between Mediterranean Diet score, diagnostic subtype, and stroke severity index. Atherosclerosis. 2015;243(1):260‐267. 10.1016/j.atherosclerosis.2015.09.017 26409625

[66] Rogers MA , Lemmen K , Kramer R , Mann J , Chopra V . Internet‐delivered health interventions that work: systematic review of meta‐analyses and evaluation of website availability. J Med Internet Res. 2017;19(3):e7111. 10.2196/jmir.7111 PMC5384996 28341617

[67] Schoeppe S , Alley S , Van Lippevelde W , et al. Efficacy of interventions that use apps to improve diet, physical activity and sedentary behaviour: a systematic review. Int J Behav Nutr Phys Activ. 2016;13(1):127. 10.1186/s12966-016-0454-y PMC5142356 27927218

